# Cancer of the Rectum

**Published:** 1891-07

**Authors:** L. L. McArthur

**Affiliations:** Chicago, Ill.


### CANCER OF THE RECTUM.*

BY L. L. McARTHUR, M. D., CHICAGO, ILL.

In my brief experience as a surgeon it has been my fortune to number among my cases six examples of this disease in various stages of advancement, as follows : One, female, cancer within two inches of anus, colostomy and excision; one, female, cancer involving anus and lower rectum, colostomy, excision; one, female, cancer involving anus and rectum, colostomy, no excision; one, female, cancer high in rectum, no colostomy, curettage; one, male, Kraske's operation, colostomy (Dr. Fenger's case); one, male, no excision, currettage, no colostomy. With these there were no deaths as a result of the operations, though Kelsey states that the mortality for excision is thirty-three per cent.

Let me, before presenting the history of a case, call to your attention the following significant facts: In a careful inquiry—made by Cripps—into the family history of a large number of cases of cancer, the percentage of mortality by that disease was found almost identical with that of the general population by the same. In a very large proportion the disease is so situated that an infection could have been plausibly possible. Infection of husband from wife suffering from cancer of cervix has been in several instances well authenticated by competent observers. A much greater frequency exists near the sea than inland, and both Sheurlen, of Munich, and Thoma, of Heidelberg, have demonstrated the frequent presence of micro-organisms (psorosperms), which, in their opinion, have a specific relation to the causation of this disease.

In relative frequency of type, cylindrical-celled, flat-celled (epithelioma), and papillo-carcinoma stand in the order named. Allingham, who in 1886 had already reported thirty-nine cases of excision, is authority for the statement that rectal carcinoma

*Read before the Chicago Gynecological Society, January 16th, 1891.

usually runs its course in twenty-four months. He notes, however, cases ending in death in from four months to four years. His experience had been also (contrary to that usually noted) that the disease occurs more frequently in males than in females, and that its most usual site is within three inches of the anus. Cachexia appears at a very early period. The ages of greatest frequency range from forty-five to fifty-five, though it has been noted as early as the age of six. Cancer in this location frequently escapes the observation of the general practitioner, being mistaken for hemorrhoids; and, for this reason, too great care cannot be taken in the examination of middle-aged people in whom there are symptoms referable to the rectum.

*Treatment.*—James Adams urges that in every case there should be made a colostomy, saying: "In cases of any but the slightest degree the operation of removal may prove incomplete and the disease speedily return; . . . after complete extirpation of the lower end of the rectum the subsequent contraction is often very great, and even at times intractable, and in any case the healing of the wound will be much expedited and the chances of local recurrence diminished by diverting the course of the fecal matter."

Allingham justly condemns the making of a colostomy in every case of cancer of the rectum, stating that "often neither pain nor obstruction will ensue for months, or they may never occur and the patient may die from some other malady. Of course if a surgeon at once persuades all his patients who have malignant growths in their rectum to submit to colostomy under the promise that life will be prolonged or suffering averted, he will have many cases to report and very good but very valueless statistics."

There are four factors which make this operation justifiable: First, because the mortality of rectal excision can be immensely reduced by diverting the fecal matter from the site of the excision. Second, because there are in some of the cases excruciating pains, caused by the passage of fecal matters over the ulcerating carcinoma, which can be relieved by a colostomy, winning at the same time for the surgeon the gratitude of the patient.

Third, because in those cases in which the disease extends higher than the lower three inches, there is sure to be sooner or later a stenosis. This Jessop has demonstrated at the late meeting of the British Medical Association. He calls attention to the fact that where the disease is low down in the rectum complete obstruction seldom occurs, and that the opposite is true where the disease is high up. The reasons for this difference are to be found in the anatomy of the parts themselves; for while the rectum, as it approaches the outlet, becomes more closely applied to the sacrum and pelvic wall, in its superior portion it is comparatively free. Thus the contractile action of the colon is exerted with effect in forcing its contents through the contracted ring when that ring is fixed and immovable. But when the narrow portion is freely movable, as it is when situated in the upper portion of the rectum, the efforts of the bowel above succeed only in invaginating or otherwise displacing the growth, and fail altogether to effect any onward movement of the contents (Kelsey). Fourthly, it has been the experience of most operators that the cicatricial contraction which follows such an operation is often excessive and intractable, as in any of the inflammations, specific or simple, which so frequently result in a stenosis here. Allingham has found that if he would maintain the gut in a useful and patulous condition, it is necessary to have the patient wear a gutta-percha tube, which can be removed at will. Finally, an argument which needs no champion is the fact that thirty-three per cent. of all cases of carcinoma (as shown by the researches of Jessop in one hundred and two cases which were allowed to follow their course without any surgical interference) die from obstruction.

To sum up, I would urge that *prior to every excision in every painful case*, and in every case where the disease was situated *high up*, that a colostomy be made, the choice being in favor of the left inguinal. The method of excision recommended by the French surgeons has been that which I have utilized, preceding each excision, however, by a colostomy two or three weeks prior to the final operation. In this the main feature is a deep incision which exposes the posterior segment of the rectum from the anus to the coccyx, when it is an easy matter to dissect out the rectal tube until one comes to the anterior portion. Here, if it is found that the disease involves one or more of the coats of the vaginal wall it is, in my opinion, best to remove a longitudinal segment of the entire thickness of the same, as it both renders the operation safer and more easily accomplished, and does not, as Kelsey would infer, greatly increase the danger. When the sphincters are involved a circular incision should surround the anal opening, and all be removed together. Dr. Guerin's suggestion that the gut be cut through by the ecraseur, modified by the passing through the normal gut of several threads for purposes of fixation of the proximal end after removal, as recommended by Verneuil, has been the procedure employed in the excisions I have made. The proximal end is then to be stitched to the posterior angle of the perineal incision or to the left side of the coccyx, and, after stitching up the vaginal wound in much the same way as after a posterior colporrhaphy or laceration, deep transverse perineal stitches render the making of a new and extensive perineal body very easy.

When the type of the disease is that of the cauliflower-like growth known as papillo-carcinoma, I believe the best practice is to remove it with the curette rapidly and well down to the base of the growth. The hemorrhage, very active and easily provoked while in the soft tissues of the tumor, is easily controlled when the base has been reached. In two such cases I have been successful in for a time relieving them of their distressing symptoms, but have not been able to follow their history for more than six months after operation.

The case I now report is of interest in showing the benefit to be derived from surgical interference.

CASE.—After having suffered with what she believed to be hemorrhage, the patient came to St. Luke's Hospital a year and a half ago, with symptoms of absolute stenosis of the intestine, and requiring immediate relief. The diagnosis of carcinomatous obstruction of the rectum being made, a colostomy in the left lumbar region was done, with relief to the urgent symptoms. After the lapse of three weeks, the artificial anus being well established and healed, an incision of the rectum was practiced after the usual methods by a deep posterior incision from the anus to the left side of the coccyx well down the posterior wall of the rectum, which was then dissected laterally until the vaginal wall was reached, which was found to be involved to the level of the posterior lip of the cervix in the carcinomatous growth. The posterior wall of the vagina was removed, as well as the rectum, to this level, including the sphincter muscles. The rectum was stitched to the skin of the left side of the coccyx, and deep transverse stitches were inserted to make a new perineal body. There was speedy union and rapid convalescence. After the lapse of one year she returned to me with the artificial anus presenting a normal rectal mucuos membrane normally attached to the integument to the left side of the tip of the coccyx, with the artificial anus almost closed from surgical interference by Dr. John E. Owens, but with a return in the perirectal tissue of the original trouble to such an extent that the line of cicatrix in the vaginal wall posteriorly and in the anterior rectal wall was again invaded by the new growth, which was beginning to cause painful defecation as at first. The patient, being much frightened with the symptoms of a return, came to me requesting a repetition of the operation. This at first I refused to make, telling her that I did not believe her longevity could be increased by such a procedure nor her condition materially improved. She then consulted Drs. Parkes and Fenger, both of whom, she stated, promised to operate upon her and offered her hopes of at least temporary relief. Coming back to me with this history and the threat that if I would not operate somebody else would, I had her admitted in the last week of September, 1890, to the Michael Reese Hospital, where I excised the portion of the rectum which had been drawn down and attached to the integument at a point on a level with the posterior lip of the cervix. I dissected out laterally, in so far as I could reach, all indurated tissue. I then found that it was impossible to bring the end of the rectum down to the integument, no matter how far I might extend my posterior incision, and decided that the best thing I could do would be to suture the end of the rectum at the top of the vaginal incision after the cicatrix had been removed. I did this, then united the vaginal mucous membrane, much as is done for a laceration or operation for posterior colporrhaphy, and brought the lateral pelvic tissues together by very deep, heavy silk sutures, and, strangely enough, obtained a perfect union. The patient has, since the second week in November, been at home, is feeling well, has gained in weight, and has several times come to my office, each time stating that she feels better than she did during the year which elapsed after the first operation, that she now has control of the bowel and is capable of evacuating its contents without any artificial assistance—that is, without a douche, which I advised when she first left the hospital.

I believe this to be a unique case. I do not find, in what literature I possess, reference to a similar operation. I have no doubt that she will ultimately have a return of the trouble, because the cicatricial contraction which normally occurs with any inflammatory deposit about the rectum, whether from specific or simple inflammations, has already produced some suspicious induration.

